# Impacts of unfavourable lifestyle factors on biomarkers of liver function, inflammation and lipid status

**DOI:** 10.1371/journal.pone.0218463

**Published:** 2019-06-20

**Authors:** Ulla Nivukoski, Markus Niemelä, Aini Bloigu, Risto Bloigu, Mauri Aalto, Tiina Laatikainen, Onni Niemelä

**Affiliations:** 1 Department of Laboratory Medicine and Medical Research Unit, Seinäjoki Central Hospital and Tampere University, Seinäjoki, Finland; 2 Department of Medicine, University of Oulu, Oulu, Finland; 3 Center for Life Course Health Research, University of Oulu, Oulu, Finland; 4 Infrastructure for Population studies, University of Oulu, Oulu, Finland; 5 Department of Psychiatry, Seinäjoki Central Hospital and Tampere University, Tampere, Finland; 6 National Institute for Health and Welfare (THL), Helsinki, Finland; 7 The Institute of Public Health and Clinical Nutrition, University of Eastern Finland, Kuopio, Finland; 8 Joint Municipal Authority for North Karelia Social and Health Services, Joensuu, Finland; Universitat de les Illes Balears, SPAIN

## Abstract

**Background:**

Adopting a healthy lifestyle is associated with prolonged life expectancy. The main modifiable lifestyle-related risk factors are hazardous alcohol drinking, smoking, excess body weight and lack of physical activity. Our aim was to estimate the impact of unfavourable lifestyle factors on abnormalities in laboratory tests reflecting liver status, inflammation and lipid metabolism in a population-based cross-sectional study.

**Methods:**

The study included 22,273 participants (10,561 men, 11,712 women) aged 25–74 years from the National FINRISK Study. Data on alcohol use, smoking, body weight, and physical activity were recorded from structured interviews. The risk scores for the various life style factors were established on a 0–8 scale and used to stratify the population in classes to allow estimates of their joint effects. Serum liver enzymes (GGT, ALT), C-reactive protein (CRP) and lipid profiles were measured using standard laboratory techniques.

**Results:**

Consistent dose-response relationships were observed between the number of unfavourable risk factors and serum levels of GGT, ALT, CRP, cholesterol, HDL, LDL and triglycerides (*p* < 0.0005 for linear trend in all comparisons). When compared with those with zero risk factors, the multivariable-adjusted odds ratios (ORs) for abnormalities in all biomarkers were significantly higher in those with a sum of risk score two or more. The most striking increases in ORs in the group with the highest numbers of risk factors were observed among men in serum GGT: 26.6 (12.4–57.0), ALT: 40.3 (5.3–307.8), CRP: 16.2 (7.8–33.7) and serum triglycerides: 14.4 (8.6–24.0).

**Conclusions:**

The data support the view that the presence of unfavourable life style risk factors is associated with distinct abnormalities in laboratory tests for liver function, inflammation and lipid status. Such biomarkers may prove to be of value in the assessment of interventions aimed at reducing unfavourable risk factors and in helping individuals in long-term maintenance of lifestyle modifications.

## Introduction

Heavy alcohol drinking, smoking, excess body weight and lack of physical exercise are common modifiable risk factors of lifestyle, which may all contribute to the incidence of chronic diseases and premature death [1–5]. There may also be synergistic and additive interactions between such factors in individuals with clustering of unfavourable lifestyle factors [3, 4, 6]. Therefore, interventions aimed at reducing the number of risk factors has been recognized as an important target in both personalized medicine and public health policies [7]. Recent studies have estimated that adopting a healthy lifestyle even at the age of 50 could add more than a decade to life suggesting significant therapeutic potential for lifestyle interventions [3, 8].

A large body of evidence indicates that the occurrence of increased gamma-glutamyltransferase (GGT), and alanine aminotransferase (ALT) enzyme activities in apparently healthy individuals may often be attributed to unhealthy lifestyle factors, such as alcohol consumption or excess body weight [9–13]. The increases in these liver enzymes may also associate with extra-hepatic disease risks, including metabolic syndrome, and cardio- or cerebrovascular events [13–15]. While the biochemical pathways underlying such observations have remained unclear, previous findings have suggested that inflammatory processes [16–18], oxidative stress [19, 20] and generation of abnormal lipid profiles [21] are key pathogenic factors in the sequence of events leading to hepatotoxicity [22] or other adverse health effects, such as incident stroke [5], in individuals presenting with various clusters of risk factors.

So far, only few studies have been available to examine the individual and joint impacts of the various unfavourable life style factors on biochemical indices of health. Considering this issue, we aimed to investigate the combined effects of various lifestyle-related factors on biomarkers of liver status (ALT, GGT), inflammation (C-reactive protein) and lipid metabolism (cholesterol, HDL-cholesterol, LDL-cholesterol, triglycerides) in a large national FINRISK population-based study, which includes detailed records on alcohol consumption, smoking, physical activity and health status. It is assumed that further understanding of the biomarker behaviour in response to various types of unhealthy behaviours may improve our possibilities for interventions aimed at adopting more favourable lifestyles.

## Materials and methods

### Study design, data sources and participants

The study collects extensive data from a cross-sectional population health survey (The National FINRISK Study) carried out in Finland in 1997, 2002 and 2007. In each survey year an age- and gender stratified random sample was drawn from the population register according to an international protocol [23]. Clinical examinations included physical measurements, laboratory tests and detailed questionnaires gathering information on current health status, alcohol intake, diet, smoking, physical activity, medical history and socioeconomic factors [23, 24]. Body weight and height were measured to the nearest 0.1 kg and 0.1 cm, respectively. Body mass index (BMI, kg/m^2^) was calculated as a measure of relative body weight. The data was available from 22,273 apparently healthy individuals: 10,561 men and 11,712 women (mean age 49 ± 13 years, range 25–74 years) who completed the questionnaires and attended the medical examination. The study excluded individuals with any apparent clinical signs of liver disease, ischaemic heart or brain disease or active infection at the time of blood sampling.

The questionnaire used here for registering information on health and lifestyle has been previously developed and validated for use in international population-based health surveys [23–25]. The responses to each question on alcohol consumption, smoking, physical activity and coffee consumption are assigned to mutually exclusive and collectively exhaustive categories [25]. Data on alcohol consumption was registered from the past 12 months prior to blood sampling and included information on the types of beverages consumed as well as the amounts and frequencies of consumption. The ethanol content in different beverages was quantitated in grams of ethanol based on defined portion sizes as follows: regular beer 12 grams (1/3 L), strong beer 15.5 grams (1/3 L), long drink 15.5 grams (1/3 L), spirit 12 grams (4 cL), wine 12 grams (12 cL) and cider 12 grams (1/3 L). Information on smoking habits was collected with a set of standardized questions and the data was expressed as the amounts of cigarettes per day. Habitual physical activity including both the number and total time used for physical exercises were also registered from each participant. Coffee consumption was assessed with a set of standardized questions and expressed as the intake of standard servings of coffee (cups) per day.

The data obtained from the questionnaires was subsequently used to define scores for low risk (= 0), medium risk (= 1) and high risk (= 2) categories for each individual risk factor following recent work on health-related risk assessment in relation to alcohol consumption, smoking, BMI status and physical activity [3, 8, 26–28]. In this work, the variables were, however, categorized into three ordinal levels to yield increased statistical power as compared to previously used dichotomous classification [3]. For alcohol consumption the scores were defined as follows: 0 = no consumption; 1 = alcohol consumption between 1–14 (men) or 1–7 (women) standard drinks per week; 2 = alcohol consumption exceeding 14 drinks (men) or 7 drinks (women) per week. For smoking 0 = no smoking, 1 = 1–19 cigarettes per day, 2 = ≥ 20 cigarettes per day; for BMI 0 = BMI < 25; 1 = BMI ≥ 25 and < 30; 2 = BMI ≥ 30. For physical activity 0 represents those with physical activity over 4 hours per week; 1 = those with physical activity between 0.5 and 4 hours per week and 2 = those with physical activity less than 30 min/week. The sum of these scores provided a total number of risk factors, with higher scores (maximum = 8) indicating an unhealthier lifestyle.

The approval for the data collection was received from the Coordinating Ethics Committee of the Helsinki and Uusimaa Hospital District in 2002 and 2007 and from the Ethics Committee of the National Public Health Institute in 1997. All surveys were conducted in accordance with the Declaration of Helsinki according to the ethical rules of the National Public Health Institute.

### Laboratory analyses

Serum liver enzymes (ALT and GGT) were measured by standard clinical chemical methods on an Abbott Architect clinical chemistry analyzer following the recommendations of the assay manufacturer (Abbott Laboratories, Abbott Park, IL, USA). High-sensitivity CRP, a biomarker of inflammation, was determined using a latex immunoassay (Sentinel Diagnostics, Milan, Italy) with the Abbott Architect c8000 clinical chemistry analyzer. Lipid profiles included determinations of total cholesterol, high-density lipoprotein-associated cholesterol (HDL), low-density lipoprotein (LDL) and total triglycerides using standard enzymatic methods. All laboratory tests were subjects to continuous external quality control programs organized by Labquality, Finland and CDC (Center for Disease Control and Prevention) quality assurance and standardization program for serum lipids. The cut-offs for the normal limits of the different markers were as follows: ALT (50 U/L men; 35 U/L women), GGT (60 U/L men; 40 U/L women), CRP (3.0 mg/L), cholesterol (5 mmol/L), HDL cholesterol (1.0 mmol/L men, 1.2 mmol/L women), LDL cholesterol (3.0 mmol/L), triglycerides (1.7 mmol/L).

### Statistical methods

The main characteristics were examined using analysis of variance (ANOVA) with polynomial contrasts to reveal possible trends across increasing risk score categories. The distribution of abnormal biomarker levels across the risk categories were analysed by chi-square test for trend. Binary logistic regression was used to estimate the odds ratios (ORs) of abnormal biomarker levels associated with the risk score categories, adjusting for age and coffee consumption, as these factors are known to potentially associate with abnormal biomarker levels and showed association in univariate analysis. All factors were entered simultaneously into the multivariable model. Potential multicollinearity among the covariates was examined by calculating the Variance Inflation Factor (VIF) and no evidence was found. Correlations between the risk scores and various biomarkers were calculated using Spearman’s rank correlation coefficients. The analyses were carried out with IBM SPSS Statistics 24.0 (Armonk, NY: IBM Corp.). A *p*-value < 0.05 was considered statistically significant.

## Results

The main demographic characteristics of the participants classified to subgroups according to the distribution of unfavourable lifestyle risk factor scores and gender are summarized in Table 1. Higher levels of alcohol consumption, increased body weight, smoking and physical inactivity were found to characterize the individuals with high risk scores. Age of the participants showed a quadratic association between the risk scores such that the highest mean ages were noted in the middle portion of the risk score categories (*p* < 0.0005 for both genders). Coffee consumption was found to increase with increasing number of risk factor scores in both men and women (*p* < 0.0005 for linear trend in both genders).

**Table 1:** Main characteristics of the participants, as classified according to lifestyle risk factor scores.

Men								
Risk score	0	1	2	3	4	5	6	7–8
n (%)	217 (2.1)	1131 (10.7)	2321 (22.0)	2737 (25.9)	2181 (20.7)	1213 (11.5)	563 (5.3)	198 (1.9)
Age, years, mean ± SD	44.1 ± 14.3	47.5 ± 14.3	50.0 ± 14.3	51.2 ± 13.5	50.1 ± 13.2	49.3 ± 12.2	47.9 ± 11.7	47.4 ± 10.4
Alcohol consumption, g/day	0.0 ± 0.0	4.4 ± 6.3	6.9 ± 8.8	10.2 ± 13.0	15.3 ± 17.6	22.9 ± 25.1	33.0 ± 30.0	41.9 ± 29.8
BMI	23.1 ± 1.4	24.1 ± 2.1	25.6 ± 2.8	27.2 ± 3.3	28.6 ± 4.4	29.3 ± 4.7	29.7 ± 5.1	31.7 ± 4.1
Waist circumference, cm	82.8 ± 5.7	86.9 ± 7.0	91.3 ± 8.7	96.2 ± 9.9	100.1 ± 12.2	102.1 ± 12.6	103.1 ± 13.2	108.4 ± 11.1
Smoking, cigarettes/day	0.0 ± 0.0	0.3 ± 1.8	1.0 ± 3.4	2.7 ± 6.2	5.8 ± 8.9	11.4 ± 11.0	18.2 ± 12.0	24.3 ± 9.4
Coffee, cups/day	3.6 ± 2.9	3.9 ± 2.7	4.0 ± 2.7	4.5 ± 3.0	5.0 ± 3.5	5.3 ± 3.6	5.7 ± 3.7	5.9 ± 4.4
Physical activity, number of exercises per week	4.3 ± 2.6	3.5 ± 2.1	2.9 ± 2.0	2.4 ± 2.0	2.0 ± 2.2	1.4 ± 1.7	1.3 ± 2.1	0.9 ± 1.8
Women								
Risk score	0	1	2	3	4	5	6	7–8
n (%)	447 (3.8)	1939 (16.6)	3183 (27.2)	3004 (25.6)	1945 (16.6)	816 (7.0)	297 (2.5)	81 (0.7)
Age, years, mean ± SD	41.5 ± 12.5	44.8 ± 13.4	47.8 ± 13.5	49.5 ± 13.2	49.9 ± 13.1	48.8 ± 12.2	47.5 ± 11.0	48.4 ± 11.4
Alcohol consumption, g/day	0.0 ± 0.0	1.9 ± 3.2	3.3 ± 5.0	4.6 ± 7.5	6.4 ± 8.0	12.3 ± 11.9	16.8 ± 15.2	22.8 ± 18.7
BMI	22.4 ± 1.6	23.0 ± 2.4	24.7 ± 3.3	27.4 ± 4.9	29.9 ± 5.7	30.7 ± 6.0	31.0 ± 5.7	33.3 ± 4.7
Waist circumference, cm	73.8 ± 5.8	75.7 ± 7.3	79.7 ± 9.1	86.3 ± 12.2	92.4 ± 14.0	94.6 ± 14.5	95.9 ± 14.0	102.1 ± 12.0
Smoking, cigarettes/day	0.0 ± 0.0	0.2 ± 1.2	0.9 ± 3.1	1.9 ± 4.6	3.6 ± 6.2	6.8 ± 8.3	14.6 ± 11.0	18.8 ± 6.7
Coffee, cups/day	3.1 ± 2.5	3.2 ± 2.4	3.5 ± 2.4	3.8 ± 2.4	4.0 ± 2.6	4.2 ± 2.9	4.9 ± 3.1	4.5 ± 2.9
Physical activity, number of exercises per week	3.7 ± 1.8	3.2 ± 2.1	2.7 ± 2.1	2.4 ± 2.0	2.0 ± 2.0	1.7 ± 1.9	1.3 ± 2.0	0.8 ± 0.8

BMI, body mass index; n, number of observations

**Fig 1:**
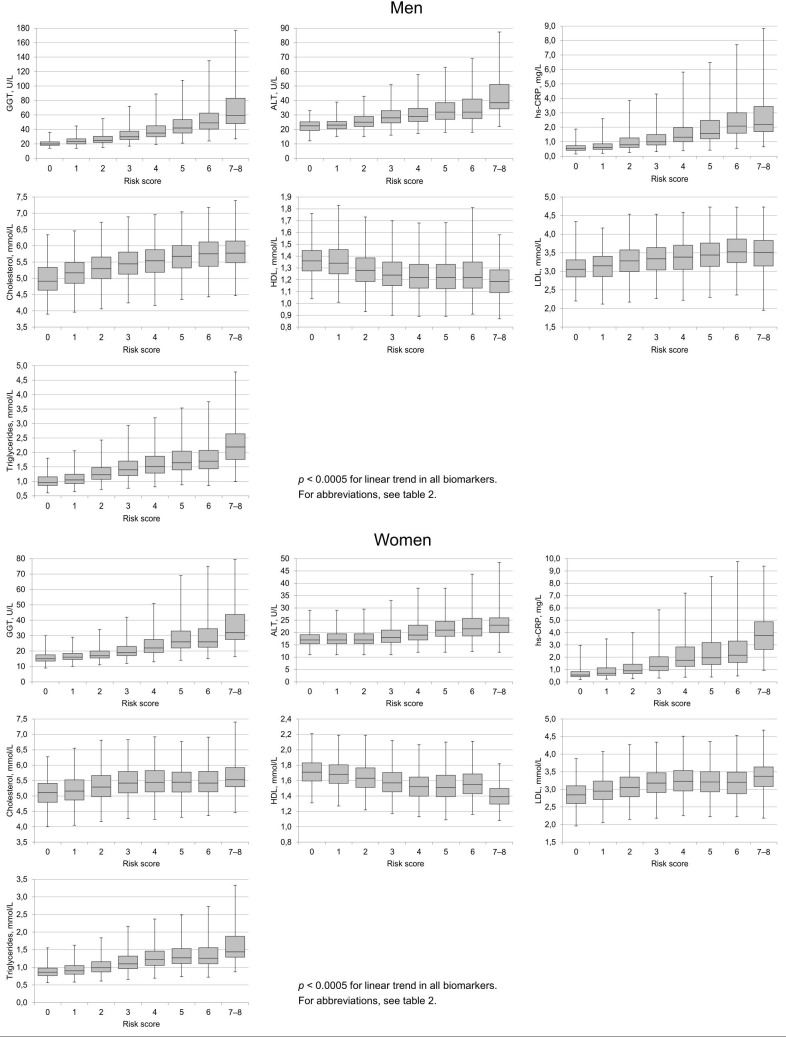
Biomarkers of liver function, inflammation and lipid status in individuals with varying lifestyle risk factor status. The data for liver enzymes (GGT, ALT), hs-CRP (biomarker for inflammation) and lipid profiles (cholesterol, HDL, LDL, triglycerides) are shown for both men and women as medians and interquartile ranges. The box represents the middle 50% of the values and the whiskers go down to the 10^th^ percentile and up to the 90^th^. The scores for the individual risk factors were defined as follows: Alcohol consumption, 0 = no consumption; 1 = alcohol consumption between 1–14 (men) or 1–7 (women) standard drinks per week; 2 = alcohol consumption exceeding 14 drinks (men) or 7 drinks (women) per week Smoking, 0 = no smoking, 1 = 1–19 cigarettes per day, 2 = ≥ 20 cigarettes per day BMI, 0 = BMI < 25; 1 = BMI ≥ 25 and < 30; 2 = ≥ 30 Physical activity, 0 = physical activity over 4 hours per week; 1 = physical activity between 0.5 and 4 hours per week; 2 = physical activity less than 30 min per week. The sum of the above scores provided a total number of risk factors, with higher scores (maximum = 8) indicating an unhealthier lifestyle.

Fig 1 demonstrates the median and interquartile ranges for the various biomarkers in groups with different risk factor status. Consistent dose-response relationships were observed between the number of unfavourable risk factors and biomarker levels in all biomarkers. The frequencies of values exceeding the upper normal limits for GGT, ALT, CRP and triglycerides or deviations from the target ranges for serum lipids in the different subgroups are summarized in Table 2. The occurrence of abnormal findings in each laboratory parameter was found to increase in a rather linear and significant manner as a function of the risk score status (*p* < 0.0005 for all comparisons).

**Table 2:** The proportion (%) of abnormal biomarker findings in individuals classified according to the number of life-style associated risk factor scores.

Men									
Risk score	0	1	2	3	4	5	6	7–8	*p*^*a*^
GGT ≥ 60 U/L	3.7	5.8	8.2	14.7	20.8	29.2	38.2	49.5	< 0.0005
ALT ≥ 50 U/L	1.4	2.8	6.0	11.3	14.7	18.8	25.3	31.8	< 0.0005
CRP-hs ≥ 3 mg/L	4.2	8.9	13.3	16.0	22.0	28.0	33.8	42.1	< 0.0005
Cholesterol ≥ 5 mmol/L	47.0	57.0	64.1	69.0	69.4	73.6	74.7	82.3	< 0.0005
HDL ≤ 1 mmol/L	7.8	9.7	15.9	19.0	21.2	21.0	21.6	25.3	< 0.0005
LDL ≥ 5 mmol/L	52.9	56.3	61.4	64.8	66.3	68.0	73.5	66.9	< 0.0005
Triglycerides ≥ 1.7 mmol/L	12.0	16.8	25.7	35.6	41.4	47.8	50.3	65.7	< 0.0005
Women									
Risk score	0	1	2	3	4	5	6	7–8	*p*^*a*^
GGT ≥ 40 U/L	5.8	4.7	7.1	11.5	16.4	25.5	30.6	33.3	< 0.0005
ALT ≥ 35 U/L	3.7	5.8	6.0	9.0	12.7	14.2	16.4	11.1	< 0.0005
CRP-hs ≥ 3 mg/L	9.7	12.0	15.1	23.0	33.3	35.8	39.4	58.0	< 0.0005
Cholesterol ≥ 5 mmol/L	54.9	56.9	62.5	67.1	68.9	67.5	67.0	76.5	< 0.0005
HDL ≤ 1.2 mmol/L	6.3	8.9	12.0	16.4	22.1	23.4	20.1	30.0	< 0.0005
LDL ≥ 5 mmol/L	44.5	47.3	53.5	58.8	60.7	60.3	59.9	71.2	< 0.0005
Triglycerides ≥ 1.7 mmol/L	6.7	8.9	12.7	19.7	25.2	29.0	31.0	43.2	< 0.0005

^a^, *p* for linear trend

GGT, gamma-glutamyltransferase; ALT, alanine aminotransferase; CRP, C-reactive protein; HDL, high-density lipoproteins; LDL, low-density lipoprotein

Table 3 summarizes the multivariable relative risks of abnormal biomarker findings according to different risk categories. The biomarkers of liver status, inflammation and lipid profiles were all found to react to life-style associated risk factors in a sensitive manner and to show significant associations with the number of risk scores when compared with participants with zero risk factors. The most striking increases in ORs in the group with the highest numbers of risk factors were observed for men in serum GGT: 26.6 (12.4–57.0), ALT: 40.3 (5.3–307.8), CRP: 16.2 (7.8–33.7) and serum triglycerides: 14.4 (8.6–24.0). When using BMI as a covariate in the binary logistic regression analyses, similar findings on ORs for abnormal biomarker status were observed, except for the lack of significance for HDL cholesterol in men and for HDL-, LDL- and total cholesterol in women (data not shown).

**Table 3:** Odds ratios (ORs) for abnormal biomarker status according to individual lifestyle risk factor scores, as adjusted for age and coffee consumption.

		Men	Women
Risk score		OR (95% CI)	OR (95% CI)
GGT	0		
1		1.5 (0.7 to 3.1)	0.7 (0.4 to 1.1)
2		2.1 (1.0 to 4.4)^a^	1.0 (0.6 to 1.5)
3		4.2 (2.1 to 8.7)^c^	1.6 (1.1 to 2.4)^a^
4		6.7 (3.3 to 13.7)^c^	2.4 (1.6 to 3.7)^c^
5		10.5 (5.1 to 21.5)^c^	4.6 (3.0 to 7.1)^c^
6		16.6 (8.0 to 34.4)^c^	6.6 (4.1 to 10.6)^c^
7–8		26.6 (12.4 to 57.0)^c^	7.0 (3.8 to 13.1)^c^
ALT	0		
1		2.1 (0.3 to 16.4)	1.6 (0.7 to 3.8)
2		5.0 (0.7 to 37.2)	1.6 (0.7 to 3.8)
3		11.3 (1.5 to 82.4)^a^	2.6 (1.1 to 6.0)^a^
4		15.6 (2.1 to 114.4)^b^	3.8 (1.6 to 8.8)^b^
5		20.8 (2.8 to 153.0)^b^	4.4 (1.8 to 10.4)^b^
6		30.0 (4.0 to 222.4)^b^	5.4 (2.1 to 14.1)^c^
7–8		40.3 (5.3 to 307.8)^c^	3.5 (0.8 to 15.0)
CRP	0		
1		2.0 (1.0 to 4.0)	1.2 (0.9 to 1.8)
2		3.0 (1.5 to 5.8)^b^	1.6 (1.2 to 2.3)^b^
3		3.6 (1.8 to 7.1)^c^	2.7 (2.0 to 3.8)^c^
4		5.6 (2.8 to 11.0)^c^	4.7 (3.3 to 6.5)^c^
5		7.9 (4.0 to 15.7)^c^	5.4 (3.8 to 7.6)^c^
6		11.1 (5.5 to 22.2)^c^	6.6 (4.4 to 9.8)^c^
7–8		16.2 (7.8 to 33.7)^c^	13.7 (7.9 to 23.7)^c^
Chol	0		
1		1.4 (1.0 to 1.9)^a^	0.9 (0.7 to 1.1)
2		1.8 (1.4 to 2.4)^c^	1.0 (0.8 to 1.2)
3		2.1 (1.6 to 2.8)^c^	1.1 (0.9 to 1.4)
4		2.2 (1.7 to 3.0)^c^	1.2 (1.0 to 1.5)
5		2.8 (2.1 to 3.8)^c^	1.2 (0.9 to 1.5)
6		3.0 (2.1 to 4.2)^c^	1.2 (0.9 to 1.7)
7–8		4.9 (3.1 to 7.8)^c^	1.9 (1.1 to 3.4)^a^
HDL	0		
1		1.3 (0.7 to 2.1)	1.5 (1.0 to 2.3)
2		2.2 (1.3 to 3.6)^b^	2.1 (1.4 to 3.1)^c^
3		2.7 (1.7 to 4.5)^c^	2.9 (2.0 to 4.4)^c^
4		3.2 (1.9 to 5.3)^c^	4.2 (2.8 to 6.4)^c^
5		3.2 (1.9 to 5.3)^c^	4.7 (3.0 to 7.1)^c^
6		3.3 (1.9 to 5.7)^c^	3.9 (2.4 to 6.4)^c^
7–8		4.1 (2.3 to 7.5)^c^	6.6 (3.5 to 12.2)^c^
LDL	0		
1		1.1 (0.8 to 1.6)	1.0 (0.7 to 1.3)
2		1.4 (1.0 to 1.9)	1.1 (0.9 to 1.4)
3		1.5 (1.1 to 2.2)^a^	1.3 (1.0 to 1.7)^a^
4		1.6 (1.2 to 2.3)^b^	1.4 (1.1 to 1.9)^b^
5		1.8 (1.3 to 2.6)^b^	1.4 (1.1 to 1.9)^a^
6		2.3 (1.5 to 3.4)^c^	1.4 (1.0 to 2.1)
7–8		1.7 (1.1 to 2.9)^a^	2.5 (1.3 to 4.8)^b^
Trigl	0		
1		1.5 (0.9 to 2.3)	1.2 (0.8 to 1.8)
2		2.5 (1.6 to 3.8)^c^	1.6 (1.1 to 2.4)^a^
3		3.9 (2.6 to 6.0)^c^	2.7 (1.8 to 4.0)^c^
4		5.1 (3.3 to 7.9)^c^	3.8 (2.5 to 5.5)^c^
5		6.7 (4.4 to 10.4)^c^	4.8 (3.2 to 7.3)^c^
6		7.6 (4.8 to 11.9)^c^	5.8 (3.7 to 9.2)^c^
7–8		14.4 (8.6 to 24.0)^c^	9.7 (5.4 to 17.4)^c^

^a^, *p* < 0.05

^b^, *p* < 0.01

^c^, *p* < 0.001. For abbreviations, see Table 2.

The strongest correlations between the numbers of various unfavourable risk factors and laboratory tests were observed for serum GGT (*r*_*s*_ = 0.381 for men; *r*_*s*_ = 0.311 for women); ALT (*r*_*s*_ = 0.252 for men; *r*_*s*_ = 0.166 for women), CRP (*r*_*s*_ = 0.308 for men; *r*_*s*_ = 0.293 for women) and serum triglycerides (*r*_*s*_ = 0.274 for men, *r*_*s*_ = 0.258 for women (*p* < 0.0001 for all comparisons).

## Discussion

The present cross-sectional observational study among a large population-based sample of individuals indicate that unfavourable lifestyle factors increase the risk for abnormalities in biomarkers for liver status, inflammation and lipid profiles in a rather linear and significant manner, which supports the view that profound health benefits could be achieved following the habits of a healthy lifestyle. According to recent observations adherence to favourable lifestyle factors significantly prolongs residual life expectancy [3] and reduces the burden of various chronic diseases [5, 26, 27]. Our data further indicates that laboratory parameters could be used as tools in patient advice and guidance during interventions aimed at achieving a more favourable lifestyle. The biomarkers chosen for the present comparisons appear to be sensitive indicators of adverse biomedical effects related to lifestyle and could therefore also be used in the follow-up of individual patients for long-term maintenance of lifestyle modifications.

Recent findings in lifestyle medicine have indicated that the main determinants for adopting a healthy life style include alcohol drinking in moderation, weight control, not smoking, and taking regular exercise [3, 6, 26, 27]. These studies have also emphasized the benefits of avoiding combinations of unfavourable risk factors, which is also in accordance with the present findings using biomarker levels as outcome measures. Previous studies on alcohol consumption as an individual lifestyle risk factor have recently concluded that regular alcohol drinking in amounts exceeding 8 standard drinks per week would lower residual life expectancy at the age of 40 years by 0.5 years, the levels of 30 drinks per week leading to a loss of 4–5 years [26–28]. In individuals with excess body weight even smaller levels of alcohol consumption increase the relative risk of hepatotoxicity, as reflected in elevated liver enzyme activities, fatty changes in the liver and increased rates of mortality due to liver cirrhosis [11, 12, 29]. Previous studies have also reported significant synergistic effect of smoking and alcohol use in increasing liver enzyme activities [30, 31].

Based on current findings lifestyle intervention could be effective when treating patients with liver problems [32–34]. However, the likelihood for a wide variety of other clinical conditions, such as heart diseases, diabetes or cancer are also significantly driven by lifestyle [3, 8, 26, 27]. Typical pathophysiological characteristics associated with lifestyle and disease risks seem to include chronic inflammation, oxidative stress and altered fatty acid metabolism [9, 18, 34]. Thus, it may be expected that systematic measurements of conventional biomarkers reflecting liver status, inflammation and lipid profiles could also offer a significant contribution to the comprehensive assessment of such patients and help in elucidating the mechanisms behind the adverse effects of various behavioural phenotypes. Previously, changes in liver enzyme activities have been shown to be associated with both hepatic and extrahepatic disease risks, including cardio- and cerebrovascular risks, deposition of triglycerides in tissues and the development of insulin resistance [10, 15, 35, 36]. Based on the present analysis which excluded individuals with clinically apparent diseases at the time of the study the biomarker responses appear to represent early changes in the sequence of events leading from risk exposure to possible disease outcomes. It should further be noted that in this material similar conclusions on a significant linear relationships between the sum of lifestyle risk factors and current biomarker levels were also reached by further exclusions of individuals with any previous history of cardiac or cerebrovascular diseases, chronic inflammatory diseases, diabetes or abnormal oral glucose test (data not shown).

Previous studies have suggested possible mechanistic links between hepatic and extrahepatic disease outcomes, as supported by findings indicating that GGT enzyme is able to fuel LDL oxidation in coronary plaques [37]. In accordance with this view, alcohol and its reactive metabolites are known to exert toxic effects virtually in all tissues and even relatively low levels of chronic drinking may increase the risk for carcinogenesis [38–40], cognitive decline [41, 42], cardiac dysfunction [43–45] and all-cause mortality [28, 46], which may also associate with abnormalities in blood lipid profiles and indices of inflammation [47–49]. Based on the present data abnormalities in serum CRP, a widely used clinical biomarker of inflammation, and lipid profiles appear to follow the burden of unfavourable risk factors and abnormalities in markers of liver function in a sensitive manner. Although CRP alone may be considered as a relatively unspecific biomarker of inflammation, previous studies have shown that CRP levels predict cardiovascular events even in individuals without any atherosclerotic manifestations or conventional risk factors [50, 51]. Evidence also suggests that CRP is an important regulator of inflammatory processes [51].

Physical inactivity and sedentary behaviour are typical characteristics of an unhealthy lifestyle and increasingly common causes of health problems across the world [3, 6, 32, 52–55]. The present biomarker-based data also underscores the benefits of physical activity as an independent and significant part of a favourable lifestyle. The individuals engaged in moderate or vigorous physical activity show significantly lower risks for biomarker abnormalities than the corresponding groups of those with low or sedentary activity even in the presence of other risk factors. The data also supports the view that physical exercise could also be used as a therapeutic approach to counteract life-style associated adverse metabolic and obesogenic effects and possibly confer long-term benefits to lifestyle-associated disease burden in general [54, 56–58]. Previously, moderate to vigorous physical activity was found to improve the degree of hepatic steatosis in fatty liver disease through reducing inflammation and oxidative stress and altering lipid metabolism even in the absence of any detectable weight reduction [34]. Interestingly, recent UK biobank based study has also concluded that physically active individuals have longer life expectancies across the different levels and indices of adiposity than those with low levels of activity [58].

Based on current data the biomarker responses to factors of lifestyle seem to be significantly driven by their joint effects. However, it should be emphasized that there may also be other types of unhealthy behaviours, such as particular dietary patterns, which may contribute to adverse health effects [3, 8, 26, 27]. Unfortunately, in this work we did not have sufficient information available on the exact compositions of the diet. Here the unfavourable lifestyle factors were, however, found to be associated with an increasing trend of coffee consumption in the high risk subgroups, which is in accordance with previous observations indicating that heavy smoking may be related with increased coffee intake [59]. Interestingly, coffee consumption has been previously shown to be associated with a reduced risk for both all-cause and cause-specific mortality [60]. Lower levels of liver-derived enzymes have also been found to occur in alcohol consumers with high levels of coffee consumption when compared to those with no coffee consumption suggesting possible hepatoprotective effects of coffee intake [12, 60].

Previous work has also emphasized the role of high-fat diets in aggravating inflammation, oxidative stress and metabolic aberrations [18–20]. High carbohydrate and processed/red meat consumption together with insufficient vegetable, fruit or vitamin intake are other important dietary components which may associate with adverse metabolic and hepatic effects [18, 26, 27, 32, 61]. Thus, the individual assessment of health risks should include considerations of the quality of the diet which may include several synergistic triggers for adverse health effects, as also previously reported from both experimental animal models [20] and human studies [12, 13, 18, 62–67]. In real life situations simultaneous adherence to several low-risk lifestyle-related factors may, however, be difficult. Thus, there is an obvious need for improved national health policies emphasizing tools for health care outcome measurements. The present findings suggest a possible expanded role for clinical laboratory information in the follow-up of patients presenting with unfavourable lifestyle risk factors.

Following previous work on lifestyle factors and health risks [3], we used BMI here as a part of the risk factor scoring system instead of using it as a covariate. This may be justified to prevent over-adjustment due to controlling for a variable which may be on a causal pathway between exposure and outcome. In this work the lack of information on the quality of the diet may further support the choice of using BMI as part of the lifestyle-related index. This approach was also supported by additional analyses using BMI as a covariate where similar conclusions were also reached on a linear relationships between the sum of lifestyle risk factors and biomarker levels, except for a lack of significance for HDL-cholesterol in men and for HDL-, LDL- and total cholesterol in women.

The strengths of this study include the large number of study subjects and a comprehensive assessment of various lifestyle risk factors together with several biomarkers. The study also included separate assessments for both genders. Nevertheless, our study has some potential limitations. Due to the observational and cross-sectional nature of the study and lack of follow-up data it is difficult to derive any causal relationships. The lifestyle factors were self-reported and thus underreporting and biased recall may occur particularly in the parameters pertaining to less socially desirable behaviours. The association between the current risk factors, the quality of the diet and biomarker responses clearly warrant future studies in large follow-up materials. Future studies are also needed to examine the effect of lifestyle factors on indices of inflammation using a wider selection of biomarkers.

Nevertheless, our study demonstrates previously unrecognized relationships between life style risk factors and biomarker abnormalities, which may prove to be useful in public health recommendations. The data also suggests a potential for using biomarker-based algorithms in a comprehensive assessment of interventions aimed at reducing the risks, which based on recent findings seem to have a major impact on life expectancies and disease outcomes.
